# Survey data on voluntary nature conservation commitments of German businesses and their perceptions towards conservation credits

**DOI:** 10.1016/j.dib.2020.106625

**Published:** 2020-12-08

**Authors:** M.S. Krause, B. Matzdorf, N. Droste

**Affiliations:** a Research Area Land Use and Governance, Leibniz Centre for Agricultural Landscape Research (ZALF), Eberswalder Str. 84, 15374 Müncheberg, Germany; b Institute of Environmental Planning, Leibniz University of Hanover, Herrenhäuser Str. 2, 30419 Hanover, Germany; c Department of Political Science, Biodiversity and Ecosystem Services in a Changing Climate, Lund University, Allhelgona kyrkogata 14, 223 62 Lund, Sweden

**Keywords:** Sustainable development, Companies, Corporate responsibility, Ecosystem services, Biodiversity, Natural capital, Willingness to pay, Social science

## Abstract

To preserve biodiversity and ecosystem services, company engagement is crucial. However, available data on manager views and perceptions regarding nature conservation in particular is rare. The presented survey data gives insights into current levels and forms of business commitments for nature conservation. The data contributes to understanding business attitudes towards voluntary conservation action and includes information about factors that influence their engagement. Moreover, the data informs about manager perceptions towards the concept of nature conservation credits and, as such, allows for an evaluation of a certified biodiversity and ecosystem services market. Importantly, the dataset contains essential company characteristics to put responses into greater context. The scope of the survey is limited to German companies from secondary and tertiary sectors. Companies were sampled through proportional stratified random sampling based on size and location. The data was collected through a self-administered online-survey, conducted in 2019. The database comprises responses of 747 companies that logged into the online system. The survey data were in part analysed through structural equation modelling for an investigation of factors that drive voluntary conservation commitments [1]. Related to this analysis, a subset of 618 companies is available that provided sufficiently completed questionnaires. Both datasets, i.e. the raw data as well as the first subset used for analysis, are hosted in the public repository Open Research Data of the Leibniz Centre for Agricultural Landscape Research (ZALF), Germany. The repository also stores all coding information as well as the questionnaire: https://www.doi.org/10.4228/ZALF.DK.149. The dataset can be used, for example, by researchers from the field of environmental business management and strategy.

## Specifications Table

**Table:** 

Subject	Nature and Landscape Conservation
Specific subject area	Business behaviour and attitudes; social science; business strategy
Type of data	Tables Text documents
How data were acquired	Survey. The German questionnaire (cf. “*Questionnaire_DE*”) and the English translation of the questions and answer options (cf. “*dataset_complete_codebook*”) can be found under: https://www.doi.org/10.4228/ZALF.DK.149.
Data format	Raw Analysed
Parameters for data collection	Companies eligible for inclusion had to be located in Germany and needed to be of medium (50–249 employees) or large (250+ employees) size. Sampled companies were from diverse secondary and tertiary sectors across all federal states. Only the main company location was sampled but not individual shops or factories belonging to the same company to avoid duplicates.
Description of data collection	In total, 17,000 firms were selected through proportional stratified random sampling. Data was collected through a self-administered online survey. Selected companies were invited to participate in the survey through postal letters. Two different letter styles were tested to identify the more activating one. The letters provided a brief URL link to the online questionnaire. Participation was only possible after entering a personal participation code. Sampling and data collection was conducted in collaboration with a survey agency that facilitated the access to company addresses.
Data source location	Germany, all federal states
Data accessibility	Repository name: Open Research Data of the Leibniz Centre for Agricultural Landscape Research (ZALF), Germany Data identification number: 10.4228/ZALF.DK.149 Direct URL to data: https://www.doi.org/10.4228/ZALF.DK.149 A data preview can be found in the above stated link. The full dataset will be made available for download upon request to the authors.
Related research article	Krause, M.S., Droste, N., and Matzdorf, B. (2020). *What makes businesses commit to nature conservation?*. Business Strategy and the Environment. https://doi.org/10.1002/bse.2650[1]

## Value of the Data

• The dataset is, to the knowledge of the authors, the first data source that specifically informs about German business perceptions and actions for nature conservation. The survey focuses on targeted actions for public environmental goods and, as such, makes a valuable contribution to the environmental management literature.
• The data is valuable to those interested in understanding business responses to the loss of biodiversity and ecosystem services. Policy-makers benefit from information about factors that encourage companies to voluntarily commit to nature protection. The data is also relevant for environmental organisations and practitioners with an interest in offering certified biodiversity and ecosystem services, in support of their fundraising efforts for conservation projects.
• The data might be used to compare the results of similar studies, which focus on different countries and/ or certain business sectors or company types. Moreover, the data can be used by researchers that investigate, for instance, organisational decision-making, environmental business strategies, stakeholder management or methodological aspects of company surveys.
• The data contains information from diverse company types and, therefore, reflects the multifaceted German business landscape. In addition, the survey captured responses from companies that were already engaged for nature conservation as well as those that were not.
• With a sample size of around 700 participants, it is a large dataset suitable for a wide range of statistical analysis methods.

## 1 Data Description

Table 1 shows the available files that are stored in the Open Research Data repository, hosted by the Leibniz Centre for Agricultural Landscape Research (ZALF), Germany (data identification number: 10.4228/ZALF.DK.149, direct URL to data: https://www.doi.org/10.4228/ZALF.DK.149). Table 1 also lists the content of the following Table 2-4 of this data paper. The survey data contains information about the voluntary nature conservation commitments of German businesses in secondary and tertiary sectors. It also includes respondents’ perceptions about the concept of nature conservation credits and their willingness to pay for biodiversity and ecosystem services.

**Table 1:** List of available files in the Open Research Data repository as well as Table 2, Table 3, Table 4 of this data paper.

File name	Description	Format	Sample size
*dataset_complete*	Complete dataset of businesses which logged into the online survey system.	.csv	747
*dataset_complete_codebook*	Table containing codes of "*dataset_complete*" as well as corresponding questions in German (original language) and English (translation).	.csv	–
*questionnaire_DE*	Pdf version of the original online questionnaire in German, showing survey layout and question order. The English translation of questions and answer options is provided in the codebooks as well as in "*questionnaire_EN translated*".	.pdf	–
*questionnaire_EN translated*	English translation of the German questionnaire. To view the original survey layout, please see "*questionnaire_DE*".	.pdf	–
*subset_1*	Subset of the "*dataset_complete*" used for analysis of factors that influence nature conservation commitments of companies [1].	.csv	618
*subset_1_codebook*	Table containing codes of "*subset_1*″ as well as corresponding questions in German (original language) and English (translation).	.csv	–
*Invitation Letter_A*	Postal invitation letter template. Invitation letter A was initially sent to 1000 companies. Because it was not as successful as "*invitation letter_B*" in activating participation, it was not used for the main despatch.	.pdf	–
*Invitation Letter_B*	Postal invitation letter template. Invitation letter B was initially sent to 1000 companies. Because it was more successful than "*invitation letter_A*" in activating participation, it was used for the main despatch.	.pdf	–
*Invitation Letter_reminder*	Postal reminder letter template, which equalled the design of "*invitation letter_B*".	.pdf	–
*target_population_2017*	Secondary data retrieved from the database "Destatis" of the German Federal Statistical Office, reflecting the target population of the survey.	.csv	–
*target_population_2017_* *retrieval*	Information about how the target population data was retrieved from the Destatis database.	.txt	
Table 2	Economic sectors and their inclusion in the target population of the survey.	in data paper	
Table 3	Distribution of the target population according to size and geographical regions, based on secondary data from Destatis.	in data paper	
Table 4	Distribution of the sample frame according to size and geographical regions, based on available company addresses from the survey agency meap GmbH.	in data paper

**Table 2:** Economic sectors considered for inclusion in the survey.

Economic sectors	Target population
A – agriculture, forestry, fishing	no
B – mining	no
C – manufacturing	yes
D – energy supply	yes
E – water supply, sewage and waste disposal	yes
F – building and construction	yes
G – trade, maintenance and repair of motor vehicles	yes
H – transportation and logistics	yes
I – hospitality	yes
J – information and communication	yes
K – provision of financial and insurance services	yes
L – real estate	yes
M – provision of freelance, scientific and technical services	yes
N – provision of other economic services	yes
O – public administration, defence, social security	no
P – education and schooling	yes
Q – health and social care	yes
R – art, entertainment and recreation	yes
S – provision of other services	yes
T – provision of other mainly personal services	no
U – extraterritorial organisations and corporations	no

The complete dataset (“*dataset_complete*”) comprises 136 variables with mostly numerical codings, except text entries in 12 columns. In total, the dataset includes observations from 747 businesses that logged into the online system. The logins were tracked by the online system, based on individual participation codes that were assigned to each company address. The corresponding codebook (“*dataset_complete_codebook*”) provides coding information and related questions in German and English. Generally, binary and dummy variables were coded 1 = “yes” and 0 = “no”. Missing values were coded as 999,999 = “no answer” or 977,777 = “not applicable” due to preceding filter functions.

The questionnaire (“*Questionnaire_DE*”) shows the design of the implemented online survey. It is comprised of four main parts: 1) information about the company, 2) information about its nature conservation commitment as well as aspects influencing (non-)commitment, 3) perceptions about the concept of nature conservation credits, and 4) preferences for nature conservation credits.

A part of the survey data (“*subset_1*″) has been analysed through structural equation modelling to investigate factors that influence companies’ voluntary nature conservation commitments [1]. The subset consists of 59 variables and 618 observations. For inclusion in the analysis, the respondents needed to answer a question about whether or not their company has been engaged for the protection of biological diversity and natural habitats (cf. variable “*engaged*”). Respondents that gave no answer to this question or stated “I don't know” were excluded. The subset further differs from the complete dataset in that some variables were renamed and recoded. All information about coding, new and previous variable names as well as related questions in German and English are found in the codebook (“*subset_1_codebook*”).

The repository also provides the postal letters used to invite the companies to participate in the survey (*"Invitation Letter_A", "Invitation Letter_B"* and *"Invitation Letter_reminder"*). The files provide the template of the one-page invitation letters as well as the envelopes. The documents are provided in German.

Moreover, a secondary data table is provided, which contains information about the geographical and size distribution of the target population (*"target_population_2017″*). The data was retrieved from “Destatis”, the open source database of the German Federal Statistical Office. The table includes information based on latest available data from 2017. A detailed step-by-step guide about how the file was retrieved from the Destatis website is included as a text file in the repository (*"target_population_2017_retrieval"*).

Finally, Table 1 further includes information that is provided in the subsequent tables of this data paper.

## 2 Experimental Design, Materials and Methods

### 2.1 Target population

The target population for the business survey included companies that fulfilled the following requirements: 1) Companies with at least 50 employees: We defined this criterion because we presume that any voluntary engagement requires a certain level of flexibility and possibility in terms of finances and staff capacities, which might not be given for smaller entities. The precise threshold of 50+ employees is commonly used in official EU- or German statistical agencies to define medium-sized companies. Thus, the survey targeted medium-sized (50–249 employees) and large-sized companies (250+ employees). 2) Limitation to company headquarters: Many companies have multiple locations or run individually managed production sites. However, nature conservation engagement is often part of a wider corporate strategy [2], which tends to be worked out in headquarters rather than minor company locations. To avoid duplicates as well as contacting subordinate company locations without appropriate contact persons, we focused on company headquarters. 3) No companies from the primary sector: The survey targeted companies from diverse business sectors, except those of the agriculture, forestry, fishery or mining sector, cf. Table 2. We excluded the primary sector because due to their landscape activities nature conservation concerns are inherent to their business. The survey focused on voluntary nature conservation commitments beyond obvious operational good practice. In addition, we excluded three economic sectors from the target population as they were not suitable for our topic of interest as shown in Table 2.

These target population specifications resulted in a total population size of around 79,000 German companies, according to the German Federal Statistical Office [4]. Based on the latest available data from 2017, around 80% of the target population were medium-sized and around 20% were large-sized companies, cf. Table 3.

**Table 3:** Distribution of the target population according to size and geographical regions.

	Medium-sized companies		Large-sized companies		
German Federal State	*N*	%	*N*	%	Total
Baden-Württemberg	8,923	11.32%	2,262	2.87%	11,185
Bayern	10,236	12.98%	2,506	3.18%	12,742
Berlin	2,780	3.53%	633	0.80%	3,413
Brandenburg	1,559	1.98%	281	0.36%	1,840
Bremen	738	0.94%	178	0.23%	916
Hamburg	1,943	2.46%	520	0.66%	2,463
Hessen	4,890	6.20%	1,301	1.65%	6,191
Mecklenburg-Vorpommern	1,140	1.45%	203	0.26%	1,343
Niedersachsen	6,196	7.86%	1,256	1.59%	7,452
Nordrhein-Westfalen	13,747	17.43%	3,490	4.43%	17,237
Rheinland-Pfalz	2,713	3.44%	569	0.72%	3,282
Saarland	633	0.80%	168	0.21%	801
Sachsen	3,054	3.87%	618	0.78%	3,672
Sachsen-Anhalt	1,596	2.02%	324	0.41%	1,920
Schleswig-Holstein	1,998	2.53%	415	0.53%	2,413
Thüringen	1,669	2.12%	318	0.40%	1,987
Total	63,815	80.92%	15,042	19.08%	78,857

### 2.2 Sample frame and sampling

To gain access to company addresses of our target population we worked with the survey agency meap GmbH. This agency had access to various company databases from its cooperation with address brokers through which company contacts could be rented for the survey. From the available addresses, the survey agency created the sample frame according to the above specified criteria, cf. Table 4. In total, the agency had around 68,000 addresses available that matched the specifications of our target population. The reason that the target population and sample frame differ slightly is that not all companies which were recorded by the Federal Statistical Office were also available in the databases of address brokers. In addition, the information in the Destatis business registry referred to the year 2017, whereas the address databases might have been more or less up-to-date.

**Table 4:** Distribution of the sample frame according to size and geographical regions.

	Medium-sized companies		large-sized companies		
German Federal state	*N*	%	*N*	%	Total
Baden-Württemberg	8,246	12.11	1,864	2.74	10,110
Bayern	9,104	13.37	2,099	3.08	11,203
Berlin	2,181	3.20	441	0.65	2,622
Brandenburg	1,259	1.85	203	0.30	1,462
Bremen	607	0.89	132	0.19	739
Hamburg	1,601	2.35	470	0.69	2,071
Hessen	4,296	6.31	1,018	1.50	5,314
Mecklenburg-Vorpommern	994	1.46	149	0.22	1,143
Niedersachsen	5,270	7.74	1,064	1.56	6,334
Nordrhein-Westfalen	12,318	18.10	2,985	4.39	15,303
Rheinland-Pfalz	2,322	3.41	471	0.69	2,793
Saarland	583	0.86	160	0.24	743
Sachsen	2,648	3.89	421	0.62	3,069
Sachsen-Anhalt	1,253	1.84	201	0.30	1,454
Schleswig-Holstein	1,755	2.58	320	0.47	2,075
Thüringen	1,433	2.11	204	0.30	1,637
Total	55,870	82.07	12,202	17.93	68,072

Based on the sample frame, proportional stratified random sampling was conducted. The strata were defined according to 1) the federal states, as well as 2) the business size, differentiated by the number of employees. In total, 17,000 company contacts were sampled and invited to participate in the survey.

### 2.3 Questionnaire design

The questionnaire content was based on aspects that were found to be relevant for corporate conservation commitments, according to a preceding qualitative study [2]. Therefore, the questionnaire sought to expand and validate the findings of the qualitative study (ibid.). All in all, the questionnaire consisted of 23 to 30 questions, depending on responses to filter questions, and included mainly closed as well as some open questions. Numerous closed questions were measured on a 5-point Likert scale, which typically included an “I don't know” answer option. The survey required an estimated 15 to 20 min to complete. To ensure high quality, we invited 11 company representatives from various sectors, such as technology consultancy and tourism, to participate in a pre-test. Regarding the questionnaire design we also received consultancy from survey experts of the GESIS Leibniz-Institute for the Social Sciences in Germany.

### 2.4 Data collection

The data was collected through a self-administered online survey between August and December 2019. The online system was optimised for various mobile devices and web browsers. We invited our sampled contacts using postal invitation letters because e-mail invitations often go unread, are quickly deleted or are filtered out to the spam folder [5, 6]. One-page invitation letters addressed the respondents personally. In large companies with 250+ employees we primarily contacted the head of marketing and communication. If no such contact person was available in the database we addressed a member of the executive board or first management level. In medium-sized companies with 50–249 employees, we primarily addressed the executive manager or else a member of the first management level. We made this distinction because we presumed that executive managers in medium-sized companies tend to be involved in most decisions themselves, whereas in large or even very large corporations a lot of decision-making powers rest within the specialised departments. The available database from the survey agency meap GmbH did not include contact persons from CSR departments or environmental management, so we could not target employees in those positions.

The invitation letters contained a brief URL link and a company-specific participation code required for logging in to the survey system. They also included information and logos of the affiliated research institute as well as the sponsoring agencies, i.e. two federal ministries and one federal agency. Moreover, we assured confidentiality of the data as well as anonymity of the respondents in both the invitation letter and the login page. The letters as well as the online survey provided the name and e-mail address of the study leader. To encourage participation, we offered respondents to indicate their interest in receiving a survey results report from the finalised study, at the end of the questionnaire.

We initially designed two different versions of the invitation letter to compare the activation success: One design used colour-print, electronic signatures and friendly wording (cf. *Invitation Letter_A*), whereas the other design used a more bureaucratic style without colour-printing or signatures (cf. *Invitation Letter_B*). A despatch of both versions to 1000 companies showed a higher response success from the bureaucratically-styled letters. Therefore this design was used for contacting the remaining 15,000 companies, which was done mid-October 2019. At the end of November, a reminder letter (cf. *Invitation Letter_reminder*) was sent out to 7000 randomly selected companies that had not yet participated in the survey until then. Even though reminders are an effective way to increase response rates [7], one or numerous reminders to all non-respondents was not feasible due to budget reasons. Moreover, the identity of the companies and contact persons were not known to the research team and rested undisclosed with the survey agency so that follow-ups through e-mail or telephone were not possible.

From 17,000 postal invitations, 553 did not receive the letters due to wrong or outdated address information, bankruptcy or unavailable contact persons, e.g. due to retirement or job change. However, it is likely that the number of unavailable contact persons was higher than what has been reported back to the study team. A total of 747 participants (4.54%) logged into the online survey, cf. *dataset_complete*. A first data analysis with a subset of the survey responses [1] was done with 618 questionnaires (3.76%), cf. *subset_1*.

## Ethics Statement

We confirm that the participants in the survey have given their informed consent to participate. Participants were ensured that their participation is voluntary and that they can leave or pause the online survey at any point. It was guaranteed that any information provided is treated confidentially and anonymously.

## CRediT Statement

**Marlen Krause:** Methodology, Investigation, Formal analysis, Writing - Original Draft. **Bettina Matzdorf:** Conceptualization, Funding acquisition. **Nils Droste:** Formal analysis, Writing - Review & Editing.

## Declaration of Competing Interest

The authors declare that they have no known competing financial interests or personal relationships which have or could be perceived to have influenced the work reported in this article.
